# Hypoxia-Adaptation Involves Mitochondrial Metabolic Depression and Decreased ROS Leakage

**DOI:** 10.1371/journal.pone.0036801

**Published:** 2012-05-04

**Authors:** Sameh S. Ali, Mary Hsiao, Huiwen W. Zhao, Laura L. Dugan, Gabriel G. Haddad, Dan Zhou

**Affiliations:** 1 Department of Medicine, University of California San Diego, San Diego, California, United States of America; 2 The Center for Aging and Associated Diseases, Helmy Institute of Medical Sciences, Zewail City of Science and Technology, Giza, Egypt; 3 Department of Pediatrics, University of California San Diego, San Diego, California, United States of America; 4 Department of Neuroscience, University of California San Diego, San Diego, California, United States of America; 5 Rady Children's Hospital-San Diego, San Diego, California, United States of America; Enzo Life Sciences, Inc., United States of America

## Abstract

Through long-term laboratory selection, we have generated a *Drosophila melanogaster* population that tolerates severe, normally lethal, level of hypoxia. This strain lives perpetually under severe hypoxic conditions (4% O_2_). In order to shed light on the mechanisms involved in this adaptation, we studied the respiratory function of isolated mitochondria from the thorax of hypoxia-adapted flies (AF) using polarographic oxygen consumption while monitoring superoxide generation by electron paramagnetic resonance (EPR) techniques. AF mitochondria exhibited a significant 30% decrease in respiratory rate during state 3, while enhancing the resting respiratory rate during State 4-oligo by 220%. The activity of individual electron transport complexes I, II and III were 107%, 65%, and 120% in AF mitochondria as compared to those isolated from control flies. The sharp decrease in complex II activity and modest increase in complexes I and III resulted in >60% reduction in superoxide leakage from AF mitochondria during both NAD^+^-linked state 3 and State 4-oligo respirations. These results provide evidence that flies with mitochondria exhibiting decreased succinate dehydrogenase activity and reduced superoxide leakage give flies an advantage for survival in long-term hypoxia.

## Introduction

The complex interaction between nutrients, oxygen, and mitochondria embodies the fundamental evolutionary struggle of eukaryotic life to survive and flourish under continuous and periodic environmental challenges. For an organism to handle extrinsic challenges such as limited oxygen/nutrients supplies or intrinsic factors such as increased energy demands it has to precisely and quickly respond to a wide spectrum of stressors and modulators. Mitochondria play a central role in this paradigm through a sophisticated array of regulatory and signaling responses that are yet to be understood in detail. For example, mitochondria play unequivocal roles in the cellular and organismal response to limited supply of oxygen (hypoxia). In acute hypoxia mitochondria have been implicated as an early respondent by releasing reactive oxygen species (ROS) which in turn trigger a cascade of events involving the stabilization of hypoxia-inducible factor (HIF-1) [1], [2], [3], [4]. HIF-1 then orchestrates the transcriptional response by upregulating genes that control angiogenesis to increase oxygen delivery and by switching to anaerobic metabolism that is less O_2_-demanding [5], [6]. It appears that the HIF-1 pathway is preserved in almost every organism starting from the simplest metazoans, such as the nematode worm *Caenorhabditis elegans*, up to mammals suggesting that selective pressure for adaptation in response to variations in oxygenation was crucial throughout eukaryotic evolution.

Hypoxia contributes to a range of human diseases, such as pulmonary hypertension [7], obstructive sleep apnea [8], and ischemia/stroke [9], but it is clear from populations living at high altitude that substantial adaptation to sustained hypoxia is possible [10], [11]. It was suggested that the metabolic malleability of mitochondria under oxygen challenges is crucial for the cellular responses to a wide range of pathophysiologic oxygen tensions [12]. Less is known on the permanent changes inflicted on mitochondria by sustained hypoxia. Here we shed light on the mechanisms of mitochondria-mediated adaptation to long-term hypoxia employing a hypoxia-tolerant strain of *Drosophila melanogaster* [13], which are capable of surviving at 4% O_2_ perpetually. This hypoxia-adapted fly strain was generated through long-term laboratory selection under hypoxia pressure for more than 200 generations [13]. Phenotypic changes in this fly strain include a reduced body size and weight and a heritable trait of hypoxia tolerance, demonstrating adaptive modifications [14].

Our gene expression profiling experiments have shown recently a significant and coordinated down-regulation of genes encoding multiple metabolic enzymes, suggesting that metabolic modification played a major role in hypoxia-tolerance [15]. These genotypic changes in the adapted flies conferred an enhanced tolerance to acute hypoxia exposure as revealed by the metabolic profiles for adapted and control flies after exposure to acute hypoxia [16]. Principal Component Analysis suggested also that the adapted flies produced more ATP per glucose and created fewer protons than control flies, had lower pyruvate carboxylase flux, and had greater usage of Complex I over Complex II.

To explore these hypoxia-induced functional adaptive changes at the mitochondria level, we studied respiratory functional alterations and associated reactive oxygen species (ROS) production in isolated mitochondria from these hypoxia-adapted flies (AF). Our results confirm that the AF possess mitochondria with altered electron transport chain complexes (ETC) activity pattern that is consistent with reduced ROS production.

## Materials and Methods

### Chemicals and reagents

All chemicals were the highest available grades and purchased from Sigma Aldrich unless otherwise mentioned. DIPPMPO (5-(diisopropyl)-5-methyl-1-pyrroline-N-oxide) was purchased from Alexis Biochemicals (AXXORA, LLC., San Diego, CA).

### Isolation of mitochondria from fly thorax

Mitochondria were isolated from thoracic muscle as previously described with minor modifications [17]. Briefly, groups of 150–200 male flies were used per preparation. Live flies were chilled briefly on ice and thoraces are severed from the heads and abdomens under microscope. Isolated thoraces were placed in a chilled mortar, containing 300 µl of ice-cold isolation buffer (0.32 M sucrose, 10 mM EDTA, 10 mM Tris-HCl, 2% bovine serum albumin (BSA, fatty acid free), pH 7.3). The thoraces were pounded gently without shearing to release mitochondria, and the preparation was maintained at 0–5°C throughout subsequent washing and centrifugation procedures. The debris was filtered through Spectra/Mesh nylon (10 µm pore size), and the volume was raised to 1.5 ml by washing the nylon membrane with additional isolation buffer. After centrifugation for 2 min at 250×g, the supernatant was transferred to a new tube and centrifuged for 10 min at 2200×g. The pellet was rinsed briefly in BSA-free isolation buffer, and then resuspended in 1.5 ml of the BSA-free buffer and centrifuged again. The mitochondrial pellet was resuspended in 300 µl of BSA-free isolation buffer for the measurements of respiratory complexes I, II, III, and IV activity as well as electron paramagnetic resonance (EPR) measurement of the free radical production.

### Analysis of mitochondrial respiratory function

Oxygen consumption was measured using a Clark-type oxygen electrode (Oxygraph™, Hansatech, Norfolk, UK). Purified mitochondria from Drosophila' thorax (∼100–200 µg protein) were added to the oxymetry chamber in a 300 µl solution containing 100 mM KCl, 75 mM mannitol, 25 mM sucrose, 5 mM H_3_PO_4_, 0.05 mM EDTA and 10 mM Tris-HCl, pH = 7.4. After 2 minutes equilibration, 5 mM pyruvate and 5 mM malate were added and oxygen consumption followed for 2 minutes. ADP (250 µM) was added to measure State 3 (phosphorylating) respiration. Oligomycin (2.5 µg/ml) was added 2 minutes later to inhibit the F_0_F_1_-ATPase and determine State 4-oligo (resting) respiration. The maximal uncoupled respiratory rate was obtained by adding 0.2 µM CCCP (carbonyl cyanide m-chlorophenyl hydrazone) to the mixture. Oxygen utilization traces and rate determinations were obtained using Oxygraph™ software. Protein concentrations were quantified using the Pierce BCA microassay (Rockford, IL).

### EPR analysis of mitochondrial ROS generation

Immediately after mixing mitochondria (0.1–0.2 mg of protein) with 70 mM 5-(diisopropoxyphosphoryl)-5-methyl-1-pyrroline-N-oxide (DIPPMPO) and appropriate combinations of the substrates, the mixture was loaded into 50 µl glass capillary and introduced into the EPR cavity of a MiniScope MS200 Benchtop spectrometer maintained at 37°C. We confirmed that the detected EPR signals are substrate specific, and not due to redox cycling in the studied mixtures, by lack of signals when DIPPMPO was mixed with combinations of substrates and inhibitors in the absence of mitochondria. EPR signals accumulated over 10 min after mixing with substrates were quantified. Assignment of the observed signals from mitochondria is confirmed through computer-assisted spectral simulation using the WinSim software (http://epr.niehs.nih.gov/pest.html) and published spin parameters [18], [19]. In most cases a mixture of signals due to DIPPMPO-OOH and DIPPMPO-OH adducts, with occasional contribution from a carbon-centered radical, was detected but the complete removal of these signals upon the inclusion of SOD confirmed that superoxide radical is the exclusive source of the observed EPR-active species.

### Mitochondrial Respiratory Complex Activity

Activities of Complex I, II, III and IV were measured following previous published protocols with minor modifications [20]. Briefly, the activity of Complex I was measured by following the decrease in absorbance due to the oxidation of NADH at 340 nm with 425 nm as the reference wavelength. The mitochondrial samples were suspended in 25 mM potassium phosphate buffer (containing 5 mM MgCl_2_, pH 7.2) and subjected to three rounds of freeze/thaw. The reaction mix contains 0.13 mM NADH, 2 mM KCN, 2 µg/ml antimycin A 5 mM MgCl_2_, 2.5 mg/ml BSA, 65 µM ubiquinone1 and ±2 µg/ml rotenone in 25 mM potassium phosphate buffer, pH 7.2. The reaction mix was equilibrated at 30°C, and the changes of absorbance were monitored for 1 min to ensure that the baseline is stable. The mitochondrial sample (50 µg of protein) was added to the reaction mix to initiate the reaction, and the NADH∶ubiquinone oxidoreductase activity was measured for 3 min. Rotenone insensitive reaction was determined by adding rotenone in the reaction mix (2 µg/ml). The rotenone-sensitive NADH∶ubiquinone oxidoreductase activity was presented as the activity of Complex I. The activity of complex II was measured as succinate: ubiquinone1 oxidoreductase linked to the artificial electron acceptor DCPIP following the decrease in absorbance due to the oxidation of DCPIP at 600 nm. The reaction mix contains 20 mM succinate, 50 mM DCPIP, 2 mM KCN, 2 µg/ml antimycin A, 2 µg/ml rotenone and 5 mM MgCl_2_ in 25 mM potassium phosphate buffer, pH 7.2. The reaction mix with mitochondrial sample (50 µg of protein) was equilibrated at 30°C for 5 min. The baseline reaction rate was monitored for 3 min. The reaction is started by adding 1 µl of 65 mM ubiquinone1, and the enzyme-catalyzed reduction of DCPIP was measured for 3 min. The activity of Complex III was measured by following the increase in absorbance due to the reduction of cytochrome c at 550 nm. The reaction mix contains 1 mM n-dodecylmaltoside, 1 mM KCN, 2 mg/ml rotenone, 100 mM DBH2, 15 mM oxidized cytochrome c and 0.1% BSA in 50 mM potassium phosphate buffer, pH 7.2. After equilibration at 30°C, the reaction was started by addition of mitochondrial sample (20 mg of protein). The blank rate constant was determined by running the reaction without mitochondrial extract and subtracted from the rate constants determined for the samples. The activity of Complex IV was determined by following the decrease of absorbance at 550 nm due to the oxidation of reduced cytochrome c. The reaction mix contains 0.45 mM n-dodecyl-b-maltoside and 15 mM reduced cytochrome c in 20 mM potassium phosphate buffer, pH 7.0. The blank reaction rate was first recorded in the reaction mix without mitochondrial extract. The reaction was initiated by adding mitochondrial extract (15–20 µg of protein) and the decline of absorbance was determined for 2 min. Relative activity of each complex was presented as percentage activity normalized to the average of controls.

### Markers of Oxidative Stress

#### Lipid peroxidation

Malonaldehyde (MDA), an indicator of lipid peroxidation, was determined by LPO-586 assay kit (Oxis International Inc. Foster city, CA). In brief, Polyunsaturated fatty acid peroxides generate MDA upon decomposition, the decomposed MDA reacts with a chromogenic reagent, N-methyl-2-phenylindole, at 45°C, forms a stable chromophore and yields a maximal absorbance at 586 nm.

#### Protein oxidation

Protein carbonyl content, as a marker of protein oxidation, was determined with the OxyBlot kit (Millipore, Billerica, MA) according to the manufacturer's recommendations. In brief, carbonyl groups in the protein side chains are derivatized to 2, 4-dinitrophenylhydrazine (DNP), which can be further detected with rabbit anti-DNP antibody. Equal loading was assessed using an antibody against mouse polyclonal anti-β-actin (Sigma-Aldrich, St. Louis, MO).

#### Statistics

Data are reported as means ± standard error. Statistical analyses were carried out using OriginPro version 8.5 (OriginLab Corporation, MA, USA). Data were typically subjected to a One-Way ANOVA test followed by Tukey's post-hoc test. To statistically evaluate the differences in the overall oxygen consumption and ROS production; i.e. during both active and resting respiratory states, we employed the Two-Way ANOVA statistical comparisons using OriginPro. In this analysis, the two factors are genotype (NF vs. AF) and respiratory state (state 3 vs. State 4-oligo). Differences in the means were considered statistically significant when p<0.05.

## Results

### Suppression of oxidative phosphorylation and enhancement of resting respiration in hypoxia-adapted flies

**Figure 1:**
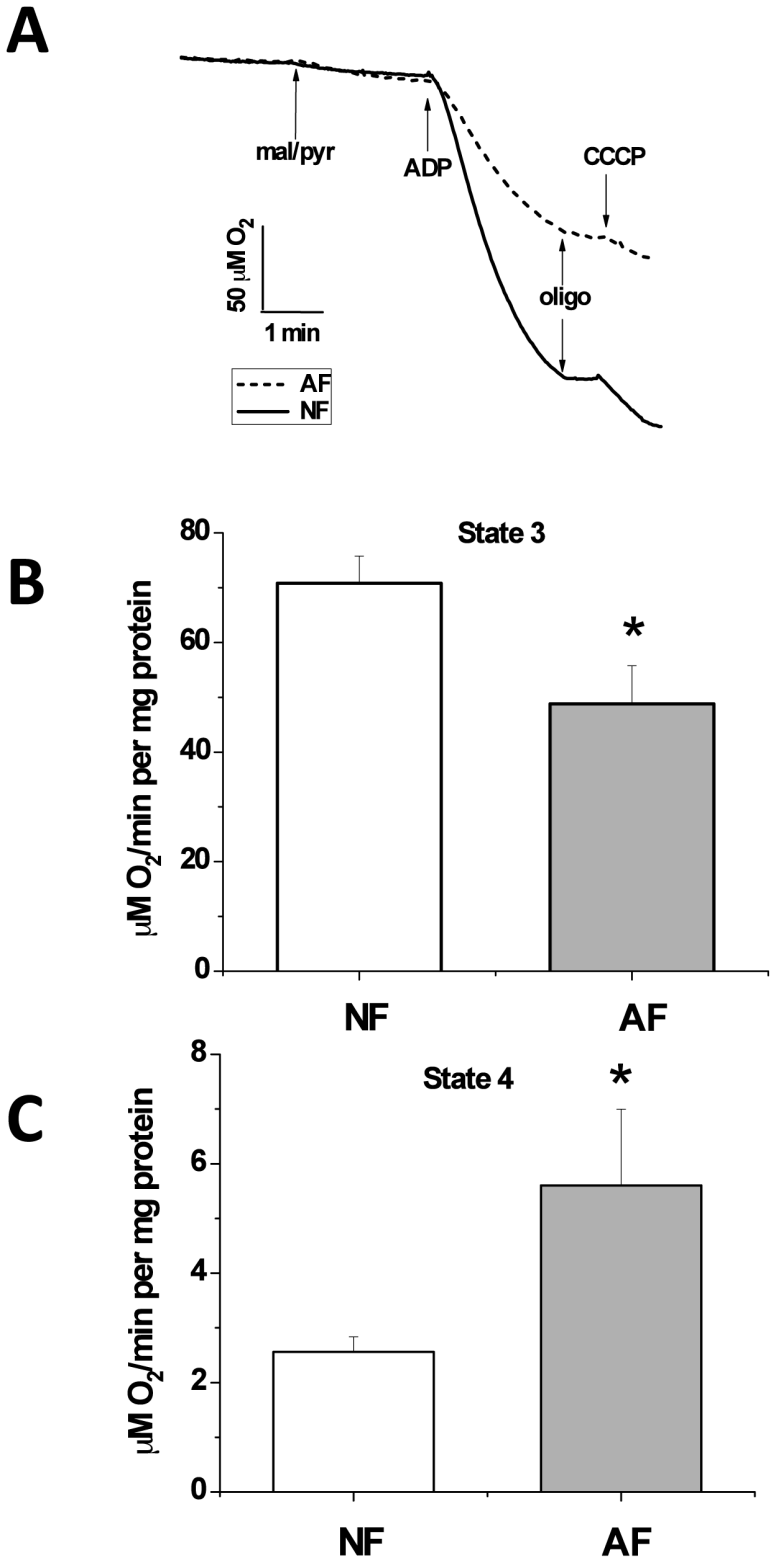
Mitochondrial respiration is depressed in hypoxia-adapted flies (AF) in comparison with normoxia-adapted flies (NF). Mitochondria were isolated from the thoraxes of ∼200 NF or AF as described (Methods), and respiratory O_2_ consumption measured using a Clark-type electrode. (A) Representative oxygen consumption traces from NF and AF mitochondria, using malate+pyruvate (5 mM each) and ADP (250 µM) to stimulate State 3 respiration, oligomycin (2.5 µg/ml) to initiate State 4-oligo respiration, and CCCP (0.2 µM) to produce maximal uncoupled respiration (State 3_U_). Analysis of oxygen consumption (µM/min/mg protein) by NF and AF mitochondria during State 3 (B) and State 4-oligo (C). State 3 respiration in AF was 31.1±14% lower than NF, p<0.05 but NF exhibited significantly faster resting (State 4-oligo) respiration (218.8±24.8% increase, p<0.05). N = 3 or 6 independent runs for AF or NF groups, respectively, with approximately 200 thoraxes utilized per run.

To examine the impact of prolonged hypoxia exposure on mitochondrial function, we followed respiratory functions of mitochondria isolated from the thoraxes of AF flies by monitoring oxygen consumption while providing them with NAD^+^-linked substrates in the presence of ADP (state 3) or while inhibiting F_1_-F_0_-ATPase by oligomycin (State 4-oligo). The results in Figure 1B indicate that the selected flies have adapted to limited oxygen supply by effectively slowing-down their mitochondrial oxidative phosphorylation during state 3 respiration (31.1±14% drop, F_(1,7)_ = 6.56, p<0.05). In contrast, AF mitochondria exhibited significantly higher resting (State 4-oligo, Fig. 1C) respiration (218.8±24.8% increase, F_(1,7)_ = 9.32, p<0.05). Although an increase in oxygen consumption during State 4-oligo respiration may appear counter-intuitive for mitochondria adapted to very limited O_2_ supply, it is important to note that the combined oxygen utilization; i.e. during both resting and phosphorylating states, by AF versus normoxia-adapted flies (NF) mitochondria is significantly reducted when statistically analyzed by a Two-Way ANOVA followed by Tukey test (25.8±12.9% drop; F_(1,17)_ = 4. 8, p<0.05). However, the accelerated State 4-oligo respiration by AF mitochondria provides an important clue that the adaptive response involves changes aiming at minimizing ROS production; see below.

### Decreased mitochondrial superoxide production in the hypoxia-adapted flies

**Figure 2:**
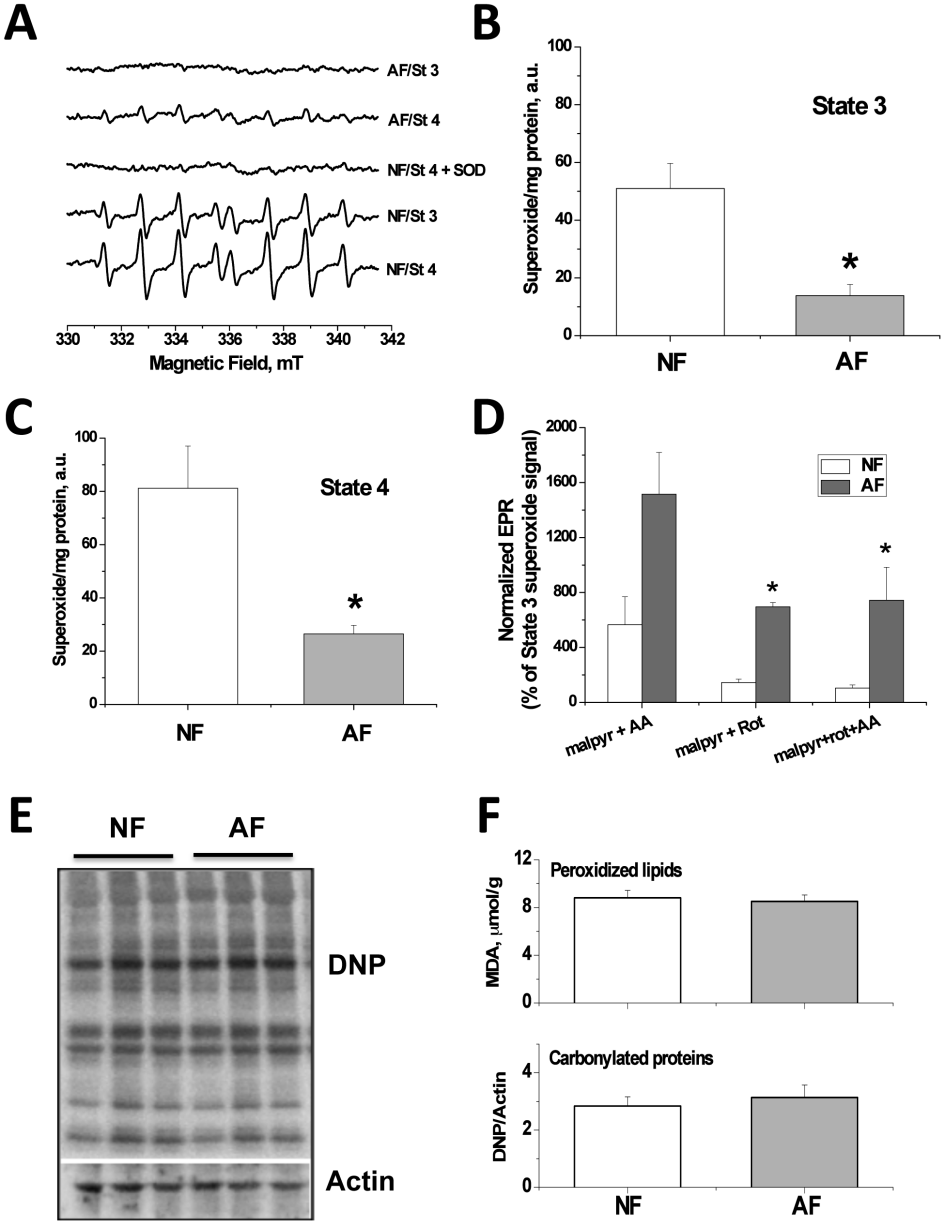
Decreased reactive oxygen species production by isolated mitochondria from AF as detected by electron paramagnetic resonance (EPR) spectroscopy. Isolated thorax mitochondria prepared as for Figure 1 were analyzed by EPR 15 minutes after addition of the spin-trap, DIPPMPO (70 mM) and mitochondrial substrates malate+pyruvate (10 mM final each) in the absence (State 4-oligo respiration ) or the presence of 500 mM ADP (state 3 respiration ). The mixture was introduced into the EPR cavity, and spectra acquired. (A) EPR spectra from NF (lower two spectra) or AF (upper two spectra) under State 3 or State 4-oligo respiration. Addition of Mn-SOD (100 U/ml) to samples in State 4-oligo respiration almost completely eliminated the signal, indicating that it is derived from superoxide (third spectrum). Quantification of the EPR signal from both NF and AF groups during State 3 (B) or State 4-oligo (C). Superoxide yield in mitochondria from hypoxia-adapted flies was 60.8% lower than that from naïve flies (F_(1,5)_ = 8.0107, p<0.05, n = 4 for control, n = 3 for hypoxia-adapted flies) during State 4-oligo, and that during state 3 was lower by 70.2% (F_(1,7)_ = 8.0107, p<0.05, n = 6 for control, n = 3 for hypoxia-adapted flies). Effects of electron transport chain complex inhibitors on mitochondrial ROS production in NF versus AF groups (D). Two markers of oxidative stress, carbonylated proteins and peroxidized lipids, are not increased in the AF group relative to the NF group (E, F). (*) indicate statistically significant difference in superoxide yield from AF relative to NF mitochondria at the given respiratory states.

In Fig. 2A, we show the EPR signals observed after 10–15 minutes incubation of mitochondria isolated from NF or AF with appropriate combinations of substrates sufficient to sustain state 3 or State 4-oligo respirations. Because exogenously added superoxide dismutase completely eliminated the EPR signal (third trace in Fig. 2A), we conclude that the observed signals are attributable mainly to hydroxyl radical that is originating from superoxide with minor contributions from lipid-derived carbon centered radical as supported by computer simulations [19].

Free radical yields from all groups (n = 4 for NF, and n = 3 for AF; approximately 200 flies per run) in both respiratory states were quantified by obtaining EPR signal amplitudes and normalizing by the total mitochondrial protein concentration in each run (Fig. 2B,C). Statistical analysis of the EPR results was carried out using a Two-Way ANOVA test of variance followed by multiple comparisons by Tukey's test. Overall, there exists a significant effect of hypoxia treatment on free radical leakage from mitochondria (F_(1,13)_ = 17.62, p = 0.002). In addition, Fig. 2 shows that: First, superoxide leakage is generally higher during resting respiration State 4-oligo (panel C) in comparison with state 3 (panel B) (statistical trends: NF, 159±12%, p = 0.061; AF, 190±44%, p = 0.46); and second, AF mitochondria produce considerably lower quantities of superoxide both during state 3 (decreased by 72.8±28%, F_(1,5)_ = 11.73, p<0.05) and State 4-oligo (decreased by 67.4±12%, F_(1,5)_ = 8.29, p<0.01). Boveris and Chance [21] have previously shown that ROS production from animal mitochondria dramatically increases during State 4-oligo.

In an attempt to identify the site(s) along the ETC where electrons are escaping to form superoxide, we performed a series of experiments that follow superoxide generation from inhibited ETC complexes by rotenone and/or antimycin A [22], [23]. In the results shown in Figure 2D, rotenone (complex I inhibitor, 10 mM) did not dramatically increase superoxide leakage during state 3 in NF (44% increase, N.S.) but increased it significantly in AF (600% increase). Complex III inhibition by antimycin A however remarkably increased superoxide yield by 5 folds in NF and by up to 14 folds in AF, an observation that is in agreement with other reports [23], [24]. Moreover, rotenone inclusion attenuated antimycin A-induced superoxide production, again in tune with the above mentioned reports and consistent with the assertion that both complex I and complex III play parts in ROS production. It is important to note however that the effects of inhibitors on ROS formation by AF mitochondria is generally higher than their effects on mitochondria from NF, analyzed under matching conditions. This appears paradoxical since AF mitochondria release significantly less superoxide in the absence of ETC inhibitors (see discussion).

We also studied two different parameters correlating with mitochondrial oxidative stress: lipid peroxidation and protein carbonylation in isolated mitochondria. Both markers did not increase in the AF mitochondria relative to the NF (Fig. 2E,F). Although increased ROS production and markers of oxidative stress under acute hypoxia exposure were consistently reported by many groups [25], [26], [27], including ours [8], mitochondria from the AF did not exhibit such increase relative to the NF. This indicates that adaptation to long-term hypoxia involves the amelioration of the initial burst of oxidative insult triggered by limited oxygen availability in the parent generation.

### Effect of hypoxia adaptation on ETC complexes activities

**Figure 3:**
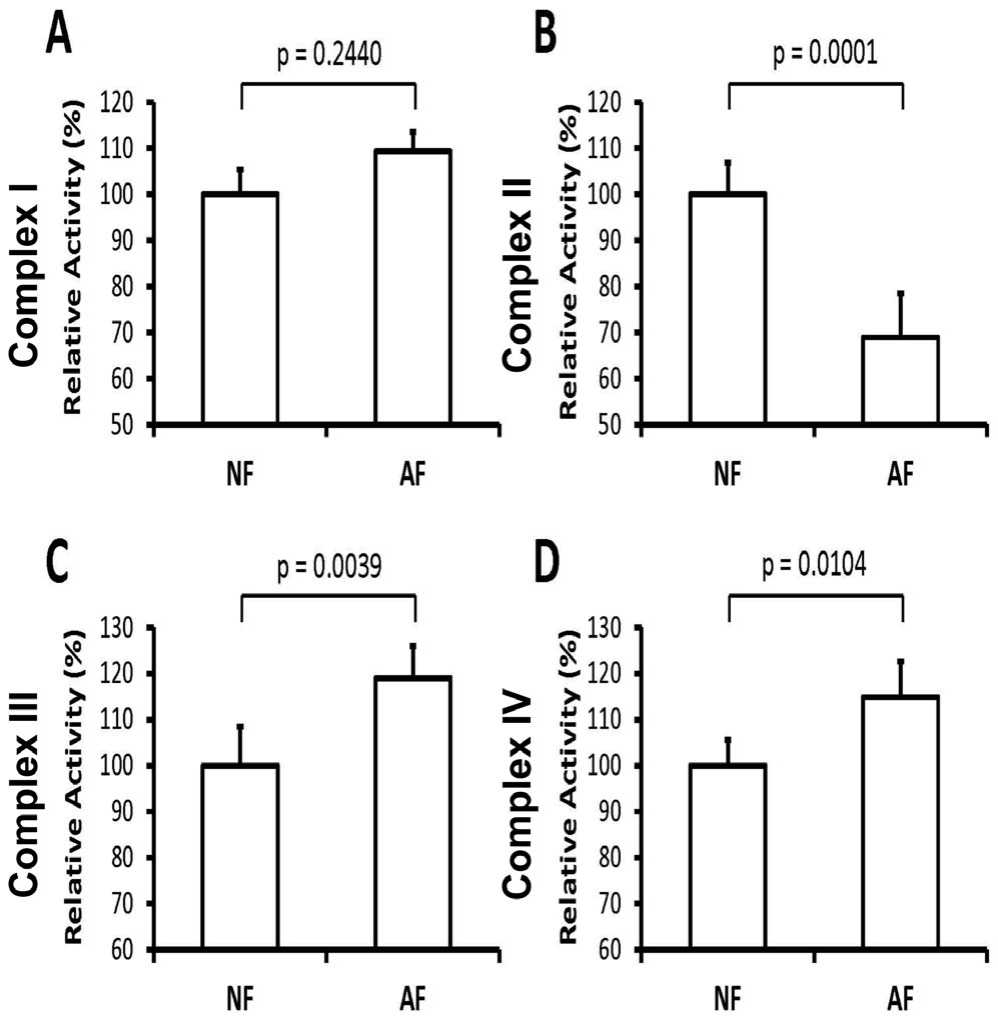
Effect of adaptation to hypoxia on mitochondrial respiratory chain complex activities. The enzymatic activity of each respiratory chain complex was measured in isolated mitochondria as described in the Methods section. (A) Complex I: no significant changes in complex activity was observed (n = 3, p>0.05) between controls (NF) and hypoxia-adapted flies (AF). (B) Complex II: significant down-regulation of complex activity was determined in complex II in the hypoxia-selected flies (AF) (n = 3, p<0.05). (C) Complex III: significant up-regulation of complex activity in the hypoxia-selected flies (AF) (n = 3, p<0.05). (D) Complex IV: significant up-regulation of complex activity in the hypoxia-selected flies (AF) (n = 3, p<0.05). All activities are presented as percent of the naive control values (mean ±SEM).

Next, we wanted to explore potential roles played by ETC individual complexes in the observed remarkable changes in metabolic utilization of oxygen and hence in ROS production. We therefore determined the activity of individual mitochondrial ETC complexes. The activity of complex II was significantly decreased by 31% (p = 0.0001) in the AF flies. In contrast, the activities of complex III and IV were increased (19% and 15% respectively, p<0.05), which indicates that the AF utilize Complex I (NADH dehydrogenase) at a higher rate, while NF rely more on Complex II (succinate dehydrogenase) activity (Figure 3).

## Discussion

To maintain oxygen homeostasis, higher eukaryotes have adopted specialized mechanisms to enhance O_2_ uptake and distribution. The resulting respiratory and circulatory systems are dynamic and capable of responding to changes in oxygen availability through an initial mitochondrial response. In fact, the ability of individual cells to sense and respond to changes in oxygen availability is critical for many developmental, physiological, and pathological processes. The results of this study demonstrate that the organismal response to long-term exposure to hypoxia, i.e. over generations, is at least partly mediated by mitochondria attempting to reduce oxygen utilization and ROS production. We investigated a population of experimentally selected *Drosophila* flies over many generations to survive a sustained 4% oxygen environment [13], [14]. We found that oxidative phosphorylation during state 3 in mitochondria isolated from thoraxes of hypoxia-adapted flies is downregulated by 30% in comparison with flies in room air. This observation is strongly supported by metabolic profiling and flux balance analysis demonstrating that adapted flies exhibit a more efficient ATP production, oxygen and substrate uptake and proton production [16]. Interestingly, downregulation of oxidative phosphorylation in AF mitochondria was associated with 220% increase in resting respiratory rate during State 4-oligo. Activity of individual electron transport complexes in AF mitochondria I, II and III were 107%, 65%, and 120% of those isolated from control flies. Again, these findings are consistent with an earlier analysis predicting that complex I activity should be greater than complex II in adapted flies [16]. Diverting the ETC entry point from complex II to complex I is known to provide a better P/O ratio and proton uptake [17]. Moreover, the decrease in complex II activity and modest increases in complexes I and III resulted in >60% reduction in superoxide leakage from AF mitochondria, both during NAD^+^-linked state 3 and State 4-oligo respirations.

It has been recognized that down-regulation of metabolism to mitigate the mismatch between supply of oxygen and demand for ATP is a systematic response to acute and chronic hypoxia [10], [11]. Under acute hypoxia the cell is forced to depend on glycolysis for ATP synthesis, which is far less efficient than mitochondrial oxidative phosphorylation [28]. Moreover, acidosis occurs as mitochondrial consumption of protons slows down and the electron transport chain complexes are generally more reduced [28], [29]. Under these conditions, leakage of electrons to oxygen to form superoxide becomes more prevalent. It is therefore likely that ROS production is an important early event in response to hypoxia, and that cell survival depends on the amelioration of ROS signaling roles; e.g. in HIF-1 pathway, as well as their detrimental roles in apoptotic and/or necrotic pathways.

Mitochondrial respiratory chain is capable of generating reactive oxygen species that account for much of the oxidative stress experienced by cells [21], [30], [31]. The levels of these ROS increase when electron flow through the respiratory chain is inhibited by respiratory inhibitors or altered by uncoupling electron transport from oxidative phosphorylation [32], [33]. Several studies have shown that exposure of cells and tissues to hypoxia increases ROS levels and oxidative stress [3], [34], [35]. This increase in oxidative stress during exposure to hypoxia depends on a functional mitochondrial respiratory chain [34]. One of the current questions is whether this increase is the result of increased generation or decreased elimination of ROS under hypoxic conditions.

During State 4-oligo resting respiration, i.e. after the ADP pool is depleted, the potential for O_2_^−•^ production increases dramatically [36]. Indeed, our results consistently showed that State 4-oligo respiration is associated with higher free radical production than state 3. Conversely, higher State 4-oligo oxygen consumption by AF mitochondria (Fig. 1C) may resemble a condition of mild uncoupling, which is known to reduce superoxide leakage, as we found (Fig. 2C). During state 3 however, superoxide, which is generated in much lower yields, reflects the basal electron leakage and its rate is expected to be a function of the rate of oxygen consumption. As a result, decreased state 3 in AF (Fig. 1B) is expected to be associated with lower superoxide leakage, as observed (Fig. 2B).

In line with the potential occurrence of ubisemiquinone at complexes I and III, we observed a substantial increase in ROS production when agents interfering with complexes I and III were applied, i.e., rotenone and antimycin A (Fig. 2D). However, rotenone (complex I inhibitor, 10 mM) did not dramatically increased superoxide leakage during state 3 in NF (44% increase, N.S.) but it increased that significantly in AF (600% increase). Complex III inhibition by antimycin A however remarkably increase superoxide yield by 5 folds in NF and by up to 14 folds in AF, an observation that is in agreement with other reports [23], [24]. Moreover, rotenone inclusion attenuated antimycin A-induced superoxide production, again in tune with the above mentioned reports and consistent with the notion that both complex I and complex III play a role in ROS production. It is important to note however that the effects of inhibitors on ROS formation in the AF mitochondria is generally higher than their effects on mitochondria from NF analyzed under matching conditions. This may appear paradoxical since AF mitochondria release significantly less superoxide in the absence of ETC inhibitors.

The fact that AF mitochondria produce less superoxide than those in NF during active oxidative phosphorylation, but more in the presence of inhibitors, indicates that AF enzymatic/non-enzymatic antioxidant levels cannot be higher than in the NF. Altenatively, it is possible that the ETC component responsible for ROS production is metabolically more efficient in AF flies (higher turn-over, lower ROS leakage with normally flowing electrons; but produces substantially higher ROS downstream when electron flow is retarded). In respiring mitochondria metabolizing malate and pyruvate, electron supply by NADH is through complex I (NADH dehydrogenase). The observation that rotenone slightly increased superoxide from the NF mitochondria suggests that complex I is not a major source of ROS in mitochondria isolated from wild type *Drosophila melanogaster*. In contrast, rotenone inhibition significantly increased superoxide signal in the AF mitochondria indicating that complex I is probably involved in ROS leakage after adaptation to hypoxia. Inhibition of complex III in both NF and AF mitochondria by antimycin A increased ROS production during oxidation of NAD^+^-linked substrates entering the ETC via complex I. Antimycin A inhibits complex III at the Q_i_ site located in the inner membrane and facing the mitochondrial matrix [37]. In the NF mitochondria, this increase by antimycin A was totally reversed in the presence of rotenone indicating that the blockade of electron flow upstream of complex III in wild type flies minimizes ROS leakage. This is consistent with the view in which complex III is the major source of superoxide in NF mitochondria. Nevertheless, the rotenone-induced partial reversal of superoxide generation by antimycin A in AF mitochondria indicates that both complexes I and III are potential leakage sites in these flies. The finding that AF mitochondria produce substantially higher superoxide concentrations in the presence of ETC inhibitors may be explained in terms of the observed increased turn-over as a result of increased activities of both complex I and complex III in these flies.

Based on published reports and on our results, here we propose the following mechanism to account for cellular and mitochondrial responses to long-term hypoxic conditions. It has been shown that complex III mediated ROS signals trigger HIF-1α stabilization in response to acute hypoxia [38], [39], [40], [41]. Under prolonged hypoxia exposure, HIF-1α is involved in the regulation of two critical adaptations that may function to prevent excessive ROS production in hypoxic cells [6], [42]. *First*, expression of PDK1 [PDH (pyruvate dehydrogenase) kinase 1] is induced. PDK1 inactivates PDH, the mitochondrial enzyme that converts pyruvate into acetyl-CoA. In combination with the hypoxia-induced expression of LDHA (lactate dehydrogenase A), which converts pyruvate into lactate, PDK1 reduces the delivery of acetyl-CoA to the tricarboxylic acid cycle, thus reducing the levels of NADH and FADH2 delivered to the electron-transport chain. This is reflected in a suppressed state 3 in the AF mitochondria. *Secondly*, the subunit composition of COX (cytochrome c oxidase) is altered in hypoxic cells by increased expression of the COX4-2 subunit, which optimizes COX activity under hypoxic conditions, and increased degradation of the COX4-1 subunit, which in turn optimizes COX activity under aerobic conditions [6]. Increased COX activity may act to dissipate excess transmembrane potential through the complete reduction of oxygen into water during resting respiration (State 4-oligo) [43], thus reducing ROS production under these conditions. These adaptations are all supported by our current results including increased complex I and IV activities on the account of complex II, suppressed overall oxygen utilization during state 3 but not State 4-oligo, and reduced ROS production from resting as well as respiring mitochondria.

In our efforts to explore metabolic response to chronic hypoxia, we have recently provided molecular mechanistic clues through metabolomic [16] and genetic [15] profiling of AF. Together with these results, our present findings suggest that survival in the hypoxia-selected flies is mediated by altered gene expression and coordinated metabolic suppression to minimize oxygen utilization and avoid ROS injuries.

## References

[1] Iyer NV, Kotch LE, Agani F, Leung SW, Laughner E (1998). Cellular and developmental control of O2 homeostasis by hypoxia-inducible factor 1 alpha.. Genes Dev.

[2] Chandel NS, McClintock DS, Feliciano CE, Wood TM, Melendez JA (2000). Reactive oxygen species generated at mitochondrial complex III stabilize hypoxia-inducible factor-1alpha during hypoxia: a mechanism of O2 sensing.. J Biol Chem.

[3] Chandel NS, Budinger GR (2007). The cellular basis for diverse responses to oxygen.. Free Radic Biol Med.

[4] Hamanaka RB, Chandel NS (2009). Mitochondrial reactive oxygen species regulate hypoxic signaling.. Curr Opin Cell Biol.

[5] Dewhirst MW, Cao Y, Moeller B (2008). Cycling hypoxia and free radicals regulate angiogenesis and radiotherapy response.. Nat Rev Cancer.

[6] Semenza GL (2007). Oxygen-dependent regulation of mitochondrial respiration by hypoxia-inducible factor 1.. Biochem J.

[7] Bosc LV, Resta T, Walker B, Kanagy NL (2010). Mechanisms of intermittent hypoxia induced hypertension.. J Cell Mol Med.

[8] Douglas RM, Ryu J, Kanaan A, Del Carmen Rivero M, Dugan LL (2010). Neuronal death during combined intermittent hypoxia/hypercapnia is due to mitochondrial dysfunction.. Am J Physiol Cell Physiol.

[9] Won SJ, Kim DY, Gwag BJ (2002). Cellular and molecular pathways of ischemic neuronal death.. J Biochem Mol Biol.

[10] Hochachka PW (1986). Defense strategies against hypoxia and hypothermia.. Science.

[11] Hochachka PW, Clark CM, Brown WD, Stanley C, Stone CK (1994). The brain at high altitude: hypometabolism as a defense against chronic hypoxia?. J Cereb Blood Flow Metab.

[12] Tormos KV, Chandel NS (2010). Inter-connection between mitochondria and HIFs..

[13] Zhou D, Xue J, Chen J, Morcillo P, Lambert JD (2007). Experimental selection for Drosophila survival in extremely low O2 environment.. PLoS One.

[14] Zhou D, Udpa N, Gersten M, Visk DW, Bashir A (2011). Experimental selection of hypoxia-tolerant Drosophila melanogaster.. Proc Natl Acad Sci U S A.

[15] Zhou D, Xue J, Lai JC, Schork NJ, White KP (2008). Mechanisms underlying hypoxia tolerance in Drosophila melanogaster: hairy as a metabolic switch.. PLoS Genet.

[16] Feala JD, Coquin L, Zhou D, Haddad GG, Paternostro G (2009). Metabolism as means for hypoxia adaptation: metabolic profiling and flux balance analysis.. BMC Syst Biol.

[17] Ferguson M, Mockett RJ, Shen Y, Orr WC, Sohal RS (2005). Age-associated decline in mitochondrial respiration and electron transport in Drosophila melanogaster.. Biochem J.

[18] Chalier F, Tordo P (2002). 5-Diisopropoxyphosphoryl-5-methyl-1-pyrroline N-oxide, DIPPMPO, a crystalline analog of the nitrone DEPMPO: synthesis and spin trapping properties.. J Chem Soc, Perkin Trans 2.

[19] Stolze K, Udilova N, Nohl H (2000). Spin trapping of lipid radicals with DEPMPO-derived spin traps: detection of superoxide, alkyl and alkoxyl radicals in aqueous and lipid phase.. Free Radic Biol Med.

[20] Kirby DM, Thorburn DR, Turnbull DM, Taylor RW (2007). Biochemical assays of respiratory chain complex activity.. Methods Cell Biol.

[21] Boveris A, Chance B (1973). The mitochondrial generation of hydrogen peroxide. General properties and effect of hyperbaric oxygen.. Biochem J.

[22] Kovacic P, Pozos RS, Somanathan R, Shangari N, O'Brien PJ (2005). Mechanism of mitochondrial uncouplers, inhibitors, and toxins: focus on electron transfer, free radicals, and structure-activity relationships.. Curr Med Chem.

[23] Chen Q, Vazquez EJ, Moghaddas S, Hoppel CL, Lesnefsky EJ (2003). Production of reactive oxygen species by mitochondria: central role of complex III.. J Biol Chem.

[24] Sugioka K, Nakano M, Totsune-Nakano H, Minakami H, Tero-Kubota S (1988). Mechanism of O2- generation in reduction and oxidation cycle of ubiquinones in a model of mitochondrial electron transport systems.. Biochim Biophys Acta.

[25] Biniecka M, Fox E, Gao W, Ng CT, Veale DJ (2011). Hypoxia induces mitochondrial mutagenesis and dysfunction in inflammatory arthritis.. Arthritis Rheum.

[26] Martin R, Fitzl G, Mozet C, Martin H, Welt K (2002). Effect of age and hypoxia/reoxygenation on mRNA expression of antioxidative enzymes in rat liver and kidneys.. Exp Gerontol.

[27] Esteva S, Pedret R, Fort N, Torrella JR, Pages T (2010). Oxidative stress status in rats after intermittent exposure to hypobaric hypoxia.. Wilderness Environ Med.

[28] Chiche J, Brahimi-Horn MC, Pouyssegur J (2010). Tumor hypoxia induces a metabolic shift causing acidosis: a common feature in cancer.. J Cell Mol Med.

[29] Gatenby RA, Smallbone K, Maini PK, Rose F, Averill J (2007). Cellular adaptations to hypoxia and acidosis during somatic evolution of breast cancer.. Br J Cancer.

[30] Cadenas E, Boveris A, Ragan CI, Stoppani AO (1977). Production of superoxide radicals and hydrogen peroxide by NADH-ubiquinone reductase and ubiquinol-cytochrome c reductase from beef-heart mitochondria.. Arch Biochem Biophys.

[31] Turrens JF (1997). Superoxide production by the mitochondrial respiratory chain.. Biosci Rep.

[32] Fridovich I (1999). Fundamental aspects of reactive oxygen species, or what's the matter with oxygen?. Ann N Y Acad Sci.

[33] Halliwell B, Gutteridge JM (1984). Oxygen toxicity, oxygen radicals, transition metals and diseases.. Biochem J.

[34] Dirmeier R, O'Brien KM, Engle M, Dodd A, Spears E (2002). Exposure of yeast cells to anoxia induces transient oxidative stress. Implications for the induction of hypoxic genes.. J Biol Chem.

[35] Grishko V, Solomon M, Breit JF, Killilea DW, Ledoux SP (2001). Hypoxia promotes oxidative base modifications in the pulmonary artery endothelial cell VEGF gene.. Faseb J.

[36] Papa S, Skulachev VP (1997). Reactive oxygen species, mitochondria, apoptosis and aging.. Mol Cell Biochem.

[37] Slater EC (1973). The mechanism of action of the respiratory inhibitor, antimycin.. Biochim Biophys Acta.

[38] Chandel NS, Maltepe E, Goldwasser E, Mathieu CE, Simon MC (1998). Mitochondrial reactive oxygen species trigger hypoxia-induced transcription.. Proc Natl Acad Sci U S A.

[39] Guzy RD, Hoyos B, Robin E, Chen H, Liu L (2005). Mitochondrial complex III is required for hypoxia-induced ROS production and cellular oxygen sensing.. Cell Metab.

[40] Mansfield KD, Guzy RD, Pan Y, Young RM, Cash TP (2005). Mitochondrial dysfunction resulting from loss of cytochrome c impairs cellular oxygen sensing and hypoxic HIF-alpha activation.. Cell Metab.

[41] Guzy RD, Sharma B, Bell E, Chandel NS, Schumacker PT (2008). Loss of the SdhB, but Not the SdhA, subunit of complex II triggers reactive oxygen species-dependent hypoxia-inducible factor activation and tumorigenesis.. Mol Cell Biol.

[42] Wigfield SM, Winter SC, Giatromanolaki A, Taylor J, Koukourakis ML (2008). PDK-1 regulates lactate production in hypoxia and is associated with poor prognosis in head and neck squamous cancer.. Br J Cancer.

[43] Campian JL, Gao X, Qian M, Eaton JW (2007). Cytochrome C oxidase activity and oxygen tolerance.. J Biol Chem.

